# Neuroprotective Effects of *Pulicaria incisa* Infusion on Human Neuroblastoma Cells and Hippocampal Neurons

**DOI:** 10.3390/antiox12010032

**Published:** 2022-12-24

**Authors:** Talya Barak, Oshrat Miller, Sarit Melamed, Zipora Tietel, Moti Harari, Eduard Belausov, Anat Elmann

**Affiliations:** 1 Department of Food Science, The Volcani Institute Center, Agricultural Research Organization, Rishon LeZion 7505101, Israel; 2 The Robert H. Smith Faculty of Agriculture, Food and Environment, The Hebrew University of Jerusalem, Rehovot 7610001, Israel; 3 Department of Food Science, Gilat Research Center, Agricultural Research Organization, Gilat 853110, Israel; 4 The Southern Arava Research and Development, Hevel Eilot 88820, Israel; 5 The Institute of Plant Sciences, Volcani Institute, Agricultural Research Organization, Rishon LeZion 7505101, Israel

**Keywords:** *Pulicaria incisa*, reactive oxygen species, Nrf2, CREB, chlorogenic acid, functional beverage, phytochemicals, neuroprotection, hippocampus, aging

## Abstract

Reactive oxygen species (ROS) and oxidative stress increase susceptibility to neurodegeneration and other age-related pathologies. We have previously demonstrated that an infusion prepared from *Pulicaria incisa* (*Pi*) has protective, anti-inflammatory, and antioxidative effects in glial cells. However, the neuroprotective activities of *Pi* infusion in cultured neurons and aging mice have never been studied. In the following study, the effects of *Pi* infusion were explored in a hydrogen peroxide (H_2_O_2_)-induced oxidative stress model in SH-SY5Y human neuroblastoma cells. Profiling of the infusion by gas chromatography–mass spectrometry identified chlorogenic acid, quercetin, and aucubin as some of its main constituents. H_2_O_2_-induced ROS accumulation and caspase 3 activity decreased SH-SY5Y viability and were prevented upon the pretreatment of cells with *Pi* infusion. Additionally, the *Pi* infusion upregulated cellular levels and the nuclear translocation of nuclear factor erythroid 2–related factor 2 (Nrf2) as well as the phosphorylation of cyclic AMP response element-binding protein (CREB). Aging mice treated daily for 18 months with *Pi* infusion exhibited reduced neuronal cell death in the hippocampus as compared to age-matched controls. We, therefore, propose *Pi* infusion as a candidate regulator of oxidative stress in the brain.

## 1. Introduction

Increased lifespans in the Western world have led to a rise in the frequencies of neurodegenerative diseases resulting from neuronal loss. Despite their increased prevalence, there are currently no effective preventive or therapeutic modalities for these deficits. Oxidative stress leading to neuronal cell death is a central process involved in the initiation and progression of neurodegenerative diseases and aging and, as such, is considered a potential target for the treatment of these diseases [1,2]. Reactive oxygen species (ROS)-elicited oxidative stress is one of the major factors involved in the aging process and may serve as one of the mechanisms underlying age-related degenerative diseases [3]. Indeed, increased ROS production damages almost every intracellular macromolecule in neurons, including proteins, lipids, and DNA, resulting in cellular dysfunction, mutations, and/or cell death. Therefore, the regulation of ROS levels is important to prevent or treat neurodegenerative diseases and aging. In recent years, natural dietary components with antioxidant activity have attracted much attention due to their role in modulating oxidative stress associated with brain aging and chronic conditions [4,5]. H_2_O_2_ is a key metabolite in oxidative stress which occurs in normal metabolism in mammalian cells at concentrations ~10 nM [6]. Higher concentrations lead to adaptive stress responses via nuclear factor erythroid 2–related factor 2 (Nrf2)/Keap1, while supraphysiological concentrations of H_2_O_2_ (>100 nM) lead to damage of biomolecules [6]. H_2_O_2_ was also shown to modulate the activity of the transcription factor cyclic AMP response element-binding protein (CREB) [7].

In a previous study, we showed that an infusion prepared from the plant *Pulicaria incisa* (Lam.) DC. (*Pi*) protected astrocytes from oxidative-stress-induced cell death, attenuated the induced ROS levels, demonstrated antioxidant properties in cell-free assays, and induced the transcription of glial-derived neurotrophic factor (GDNF) [8,9]. The infusion also triggered anti-inflammatory activity and inhibited the activation of microglial cells which secrete ROS [9]. *Pulicaria incisa* (*Pi*) is a desert plant that is used in folk medicine to treat various diseases [10,11,12,13,14,15,16,17]. Interestingly, Bedouins in Israel, northern Sinai, Egypt, and Jordan, drink infusions prepared from the wild-growing *Pi* plant in place of tea and to treat various illnesses [11,12,18]. Since *Pi* infusion possessed properties of antioxidants and protective effects in glial cells [8,9], we hypothesized that *Pi* infusion might protect neuronal cells against oxidative stress. The present study aimed to examine the antioxidative and neuroprotective effects of *Pi* infusion on H_2_O_2_-mediated oxidative stress in cultured neuronal cells and on neuronal survival in the hippocampus of aging mice. The effects of *Pi* infusion on CREB and Nrf2 activation in neuronal cells was also assessed. The presented results support the potential of *Pi* infusion to be developed as a neuroprotective antioxidant beverage.

## 2. Materials and Methods

### 2.1. Plant Material and Preparation of Pi Infusion

The *Pi* plants were grown in Southern Arava Research and Development, Hevel Eilot, Israel. Aerial parts of *Pi* plants collected in January were air-dried. The *Pi* infusion was prepared by soaking dried aerial *Pi* parts in a beaker containing boiling autoclaved double distilled water (DDW) in a ratio of 1 g plant:20 mL DDW. The beaker was allowed to cool at room temperature (RT) for 45 min, and the infusion was transferred into tubes. The tubes were centrifuged (4000 RPM, 10 min, RT), the supernatant was collected, filtered through 0.2 µm filters, and aliquots were frozen and stored at −20 °C, until use. To determine the concentration of *Pi* infusion, a 40 mL sample of the filtered supernatant was lyophilized to obtain a powder that was weighed. The average concentration of the various preparations of *Pi* infusion was ~7 mg/mL. For in vivo experiments in mice, *Pi* infusions were prepared similarly, except that sterile autoclaved tap water was used instead of DDW.

### 2.2. Chemical Characterization of Pi Infusion

#### 2.2.1. Sample Preparation

The infusion was lyophilized (Alpha-Christ, Christ, Osterode am Harz, Germany) and stored at −20 °C. Samples (30 mg) and 50 µL of 0.2 mg/mL ribitol (Sigma Aldrich, St. Louis, MO, USA) in methanol (Acros organics, Morris, NJ, USA) as internal standard, were dissolved in 0.2 mL DDW (Synergy^®^ UV Water Purification System, Merck, Darmstadt, Germany). A SEP-PAK C_18_ cartridge (Waters, Milford, MS, USA) was pre-washed with 1 mL methanol, followed by 1 mL H_2_O. The sample was loaded on the cartridge and eluted with 1.5 mL DDW. The aqueous phase was collected. Next, the cartridge was washed with 1.5 mL methanol, and the methanolic phase was collected. A 0.5 mL sample of each phase was lyophilized and then derivatized, according to an established protocol [19].

#### 2.2.2. Silylation Derivatization

Briefly, 70 µL methoxyamine hydrochloride (Sigma, Aldrich, St. Louis, MO, USA) solution (40 mg/mL in pyridine) was added to each lyophilized sample in an Eppendorf tube. Samples were then incubated in a thermoshaker for 90 min, after which, 100 µL N-methyl-*N*-(trimethylsilyl)-trifluoroacetamide (MSTFA; Sigma, Aldrich, St. Louis, MO, USA) were added. Samples were mixed in a thermoshaker at 30 °C for 30 min, and 70 µL were used for injection into GC/MS.

#### 2.2.3. Gas Chromatography–Mass Spectrometry Analysis

GC/MS analysis was performed with an Agilent 6850 GC (Agilent, Santa Clara, CA, USA), equipped with Agilent 5975C single quadrupole MS, CTC-PAL RSI 85 auto-sampler, and HP-5MS capillary column (0.25 µm × 30 m × 0.25 mm). The following conditions were applied: injector temperature 250 °C, initial temperature 50 °C for 5 min, a gradient of 5 °C/min until 180 °C, a gradient of 10 °C/min until 270 °C and a hold time of 10 min, and increasing to 320 °C. The MS parameters were set as follows: source temperature 230 °C, transfer line 325 °C, quadrupole: 150 °C, detector 325, positive ion monitoring and electron ionization (EI)-MS measurement at 70 eV [19]. Helium was used as a carrier gas, infused at 0.6 mL/min.

#### 2.2.4. Data Processing and Compound Annotation

Data were analyzed by the Unknown Analysis software (Agilent, Santa Clara, CA, USA). The percentage composition of the samples was computed from the GC peak areas. Library searches were conducted using the National Institute of Standards and Technology (NIST) 14 GC/MS Library and mass spectra from the literature. Component-relative percentages were calculated based on GC peak areas without using correction factors. Commercially available standards were used for 2-hydroxybenzoic acid, 4-hydroxybenzoic acid, genistic acid, protocatechuic acid, quininic acid, gallic acid, ferulic acid, caffeic acid, (2*R*-*E*) catechin, and quercetin (Sigma, Aldrich, St. Louis, MO, USA).

### 2.3. Cell Growth

SH-SY5Y (ATCC CRL-2266) were grown in a 1:1 mixture of Eagle’s minimum essential medium (EMEM) (ATCC, Manassas, VA USA) and F12 medium (Biological Industries, Israel), supplemented with 10% fetal bovine serum (FBS) and 1% penicillin–streptomycin (Biological Industries, Beit Haemek, Israel). Passages 12–20 were used in this study. All the experiments included a vehicle control group containing double distilled water (DDW) instead of the *Pi* infusion.

### 2.4. Measurement of Cell Viability

SH-SY5Y cells were seeded (30,000/well) in a 96-well plate and were grown for 24 h in a 1:1 mixture of EMEM: F12 medium, containing 2% FBS and 1% penicillin–streptomycin. The medium was replaced the next day with fresh medium containing different concentrations of *Pi* infusion, and cells were incubated for 2 h. Then, cells were treated with H_2_O_2_ (200 µM), and cytotoxicity was measured 20 h later. Cytotoxicity was measured using the 2,3-bis-(2-methoxy-4-nitro-5-sulfophenyl)-2H-tetrazolium-5-carboxanilide (XTT) assay, as per the manufacturer’s instructions (Sartorius AG, Goettingen, Germany). Absorbance was measured at 490 nm in a Synergy2 multi-detection microplate reader (BioTek Instruments, Inc., Winooski, VT, USA). The absorbance of the plate before the addition of XTT served as the background absorbance.

### 2.5. Quantitation of Nrf2 Levels in Nuclear Extracts

SH-SY5Y cells were seeded (10 × 10^6^/25^2^ cm flasks) and cultured overnight in a 1:1 mixture of EMEM: F12 medium, containing 2% FBS and 1% penicillin–streptomycin. The medium was replaced the next day with a fresh medium. The cells were pretreated with *Pi* infusion, after which, 200 µM H_2_O_2_ was added for an additional 2 h or 18 h. Nuclear proteins were extracted using a nuclear extraction kit (Cayman Chemical, Ann Arbor, MI, USA), according to the manufacturer’s instructions, and protein concentrations were determined using the BCA protein assay kit (Pierce Biotechnology, Rockford, lL, USA). Equal amounts (20 µg protein) from each sample were tested (27 °C in a heated block) for Nrf2 levels using the Nrf2 Transcription Factor Assay kit (Cayman Chemical, Ann Arbor, MI, USA). This assay kit detects specific transcription factor DNA-binding activity in nuclear extracts. A specific dsDNA sequence containing the Nrf2 response element is immobilized onto a 96-well plate. Nrf2 from nuclear extracts specifically binds to the Nrf2 response element and is detected by the addition of a specific primary anti-Nrf2 antibody. A secondary antibody conjugated to HRP is added to provide a sensitive colorimetric readout at 450 nm.

### 2.6. Immunocytochemistry

SH-SY5Y cells were replated (150,000 cells/well) into 24-well plates containing glass coverslips, in a 1:1 mixture of EMEM:F12 medium, containing 2% FBS and 1% penicillin–streptomycin. The medium was replaced the next day with fresh medium, and cells were then pretreated with *Pi* infusion for either 1 h or 2 h. Then, 200 µM H_2_O_2_ was added for either 2 h or 18 h. Following treatment, the cells were washed with warm Phosphate buffered saline (PBS) and then fixed with 4% formaldehyde for 15 min at room temperature, washed 3 times with PBS, and permeabilized for 3 min with 0.01% Triton-x in PBS. The cells were washed again and incubated with 10% normal mouse serum in PBS, for 30 min, at room temperature, then rinsed with PBS and incubated overnight, at 4 °C, with Alexa Fluor 488-conjugated mouse monoclonal anti-human Nrf2 antibody (sc-518033, Santa Cruz Biotechnology, Dallas, TX, USA) in PBS containing 1.5% normal mouse serum. To visualize the nuclei, the cells were washed again, stained with 4’,6-diamidino-2-phenylindole (DAPI) 1:10,000 in PBS (15 min, at room temperature), and rinsed 4 times with PBS. Images were acquired by a Leica SP8 laser scanning microscope (Leica, Wetzlar, Germany), equipped with solid-state lasers with 405 and 488 nm light, HC PL APO CS 63x/1.2 water immersion objective (Leica, Wetzlar, Germany), and Leica Application Suite X software (LASX, Leica, Wetzlar, Germany). DAPI and Alexa Fluor 488 emission signals were detected with PMT and HyD (hybrid) detectors in ranges of 415–490 and 500–550 nm, respectively. An average of 11 images/coverslip were captured, with the same exposure time for all samples. The mean fluorescence values in regions of interest were quantified with ImageJ software (NIH).

### 2.7. Determination of Caspase-3 Activity

SH-SY5Y cells were re-plated into 6-well plastic plates at a density of 2 × 10^6^ cells/2.5 mL/well in 2.5 mL of a 1:1 mixture of EMEM:F12 medium, containing 2% FBS and 1% penicillin–streptomycin. The medium was replaced the next day with fresh medium and cells were treated with *Pi* infusion, for 2 h. Then, 200 µM H_2_O_2_ was added to each well, and cells were incubated for 20 h before being scraped into tubes and washed twice with 1 mL of cold PBS (125 g, 5 min). The supernatant was discarded, and pellets were incubated in a cold lysis buffer comprised 10 mM TRIS pH 7.5, 100 mM NaCl, 1 mM EDTA, and 0.01% Triton X-100, for 30 min, on ice. After freezing in liquid nitrogen and thawing, the supernatant was centrifuged (10,000× *g*, 15 min) and the lysates were frozen at −80 °C. Protein concentration was determined using a BCA kit. Equal amounts of protein (20 µg) were tested for caspase-3 activity using the EnzChek Caspase-3 assay kit (Molecular Probes, Eugene, OR, USA). Fluorescence was measured using the Synergy^TM^ Neo2, Multi-Mode microplate reader (BioTek Instruments, Inc., Winooski, VT, USA).

### 2.8. Determination of Phospho-CREB Levels

SH-SY5Y cells were re-plated into 6-well plates at a density of 2 × 10^6^ cells/2.5 mL/well in a 1:1 mixture of EMEM:F12 medium, containing 2% FBS and 1% penicillin–streptomycin. Medium was replaced the next day with fresh medium, and cells were treated with *Pi* infusions at the indicated concentrations, for 2 h. Thereafter, 200 µM H_2_O_2_ was added for 30 min. Medium was collected into tubes and cells were washed with cold PBS and then lysed on ice in 0.5 mL/well lysis buffer containing 20 mM Tris-HCl (pH 7.5), 150 mM NaCl, 1 mM Na_2_ EDTA, 1 mM EGTA, 1% Triton x-100, 20 mM sodium pyrophosphate, 25 mM sodium fluoride, 1 mM β-glycerophosphate, 1 mM Na_3_VO_4_, 1 µg/mL leupeptin and 1 mM PMSF. After 5 min, cells were scraped and collected into tubes, frozen in liquid nitrogen, thawed, and centrifuged for 10 min at 13,000× *g*, 4 °C. The supernatant was collected and stored at −80 °C. Protein concentration was determined using the BCA kit protein assay kit (Pierce Biotechnology, Rockford, lL, USA). Equal amounts of protein were analyzed using the PathScan Phospho-CREB sandwich ELISA kit (Cell Signaling Technology, Danvers, MA, USA), according to the manufacturer’s instructions.

### 2.9. Evaluation of Intracellular ROS Levels

Intracellular ROS levels were determined using the non-fluorescent cell-permeable compound, 2’7’-dichlorofluorescein diacetate (DCF-DA; Sigma, Aldrich, St. Louis MO, USA). SH-SY5Y cells were plated onto 24-well plates (30,000 cells/0.5 mL/well) and incubated for 24 h in a 1:1 mixture of EMEM:F12 medium, containing 2% FBS and 1% penicillin–streptomycin. Cells were then labeled with DCF-DA (20 µM) for 30 min, at 37 °C. Following incubation with DCF-DA, cultures were rinsed twice with PBS which was then replaced with fresh medium. ROS levels (fluorescence) at time zero were measured in a plate reader with excitation at 485 nm and emission at 520 nm. SH-SY5Y cells were then treated with *Pi* infusions at the indicated concentrations, for 2 h, before H_2_O_2_ was added. Fluorescence intensities (indicative of ROS levels) were measured 1 h and 18 h later, by the Synergy^TM^ Neo2, Multi-Mode microplate reader (BioTek Instruments, Inc., Winooski, VT, USA). The background fluorescence intensities at time zero were subtracted from the intensities at 1 h and 18 h.

### 2.10. In Vivo Establishment of the Aging Mouse Model

The research was conducted following The National Institute of Health (NIH) guidelines for the care and use of laboratory animals and was approved by The National Permit Committee for animal science (IL-20-2-60). Twenty-seven female C57BL mice (5-week-old, weighing ~15.5 g) were purchased from Envigo, Israel. The mice were maintained under a controlled 12 h light/12 h dark cycle, with free access to food and water. The mice were randomly allocated into three groups (9 mice/group). Control mice drank only water, without *Pi* infusion. Nine mice received a daily *Pi* infusion (200 mg/kg or 600 mg/kg body weight) in the drinking water for 18 months, starting from ~1.5 months of age. The *Pi* infusions were prepared as described above, except that instead of DDW, they were prepared in sterile tap water. Each day, a new frozen tube of *Pi* infusion was thawed and then diluted into autoclaved tap water, which was added to the drinking autoclaved tap water bottles to a final volume of 60 mL. Each bottle was covered with aluminum foil to minimize exposure to light.

### 2.11. Hematoxylin and Eosin Staining Assay

At 19 months of age, mice were sacrificed, and brains were harvested and stored for 48 h in 4% formaldehyde. The tissues were processed for paraffin embedding, and serial sections (4 µM thick) were cut off with a microtome. Paraffin-embedded sections were deparaffinized and rehydrated and then stained with hematoxylin and eosin (H&E) following a previously published method [20]. After H&E staining, histopathological changes were then assessed as described in Section 2.12.

### 2.12. Digital Morphometry and Histopathological Evaluation of Pyknosis in the Hippocampus

The number of hippocampal neurons at the cornu Ammonis 3 (CA3) and dentate gyrus (DG) regions was quantified at a magnification of X20, using MATLAB software by brightness, color, and morphological-based segmentation. The image analysis system used was Image Pro Ver. 10.0.11 (Media Cybernetics, Rockwille, MD, USA). Identification of pyknosis in the hippocampus was based on the color and brightness of pixels, as measured using the ‘Smart Segmentation’ option (Machine Learning option) combined with the “Classification learning” process, which involves teaching the system morphometric and density parameters indicative of vital versus pyknotic cells [21]. This selection is saved and applied to each new image and can be changed if needed (the software keeps learning the changes). The percentage of the pyknotic neurons was calculated as the number of pyknotic neurons out of the total number of neurons (vital + pyknotic).

### 2.13. Statistical Analyses

Statistical analyses were performed with one-way ANOVA followed by Tukey–Kramer multiple comparison tests, using the JMP 7 statistical analysis software program (SAS Institute, Inc., Duxbury, MA, USA). In this analysis, columns with different letters (i.e., A, B, C) are significantly different (*p* < 0.05), and columns with the same letter are not significantly different (*p* > 0.05).

## 3. Results

### 3.1. Chemical Characterization of Pi Infusions

**Figure 1:**
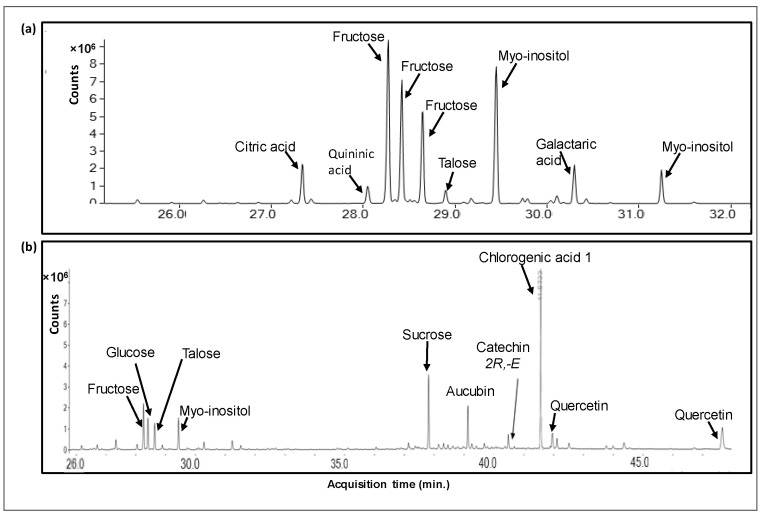
A GC/MS chromatogram of a metabolomic analysis of Pi infusion. The Pi infusion was run on an SPE Sep-Pak C18 cartridge and eluted with water to yield the aqueous phase (**a**), followed by methanol elution to yield the methanolic phase (**b**). Extracts were derivatized before analysis.

*Pi* infusions presented a rich metabolomic profile, comprised a total of 88 compounds, including 13 organic acids (accounting for 6.5% of the total peak area of the methanolic phase), 4 alcohols (0.48%), 11 amino acids (0.97%), 26 sugars and saccharides (40.4%), 3 fatty acids (1.45%), 3 terpenes (8.37%), 35 phenolic compounds (34.8%) and 5 other compounds (6.8%). The main compounds in *Pi* infusion and their relative contents in the methanolic and aqueous phases are presented in Table 1, and respective chromatograms are brought in Figure 1. In the methanolic phase, saccharides including sucrose (15.35% of total peak area), fructose (11.65%), myo-inositol (6.42%), and talose (4.52%) were most abundant, while chlorogenic acid and its derivatives (17.77%), quercetin and its derivatives (8.08%), and aucubin (2.79%) were most abundant non-saccharides. Saccharides were also the most abundant group of compounds in the aqueous phase, mainly fructose (25.91%), myo-inositol (25.90%), talose (10.31%), and glucose (9.04%), in addition to citric acid (3.81%), galactaric acid (3.57%) and malic acid (1.92%) as the prevalent non-saccharides. Our analysis revealed the presence of various chlorogenic acid isomers. Phenolic acids rarely appear in a free form, and typically are esterified with quinic or tartaric acid or a sugar moiety at different locations, giving rise to various structural isomers [22]. Specifically, chlorogenic acid is a group of caffeic and quinic acid esters, comprising six different isomers (3-O- caffeoylquinic acid (3CQA), 4CQA, 5CQA, 3,4-dicaffeoylquinic acid (3,4-DQA), 3,5-DQA and 4,5-DQA), or 26 compounds when considering *cis-trans* isomerization [23]. These have also been reported to vary in occurrence, bioavailability, and bioactivity [24]. Similarly, flavonoids, e.g., quercetin, present characteristic versatility due to structural variability, owing to conjugation with various sugar moieties at various positions [25,26]. As GC/MS metabolomic analysis cannot be used for reliable quantification, the current data only provided relative quantification of these compounds, and further use of quantitative methods, e.g., LC-QqQ-MS, accompanied by commercially available standards, would be required for supplying quantitative information about the levels of the reported metabolites in *Pi* infusion.

Metabolites were analyzed by GC/MS metabolomics following C_18_ SPE extraction with methanol (methanolic phase) and DDW (aqueous phase). Presented are metabolites that comprised at least 1% of the total ion count (total peak area) of the respective chromatograms. Quercetin and Quininic acid were identified by commercial standards. Fructose and Quercetin represent the sum of numerous derivative peaks, resulting from N-methyl-*N*-(trimethylsilyl)-trifluoroacetamide (MSTFA) derivatization at different locations of the molecules. Chlorogenic acids 1 and 2 may represent different isomers or different derivatives of the same isomer.

### 3.2. Pi Infusion Protects Neuronal Cells against Oxidative-Stress-Induced Cell Death

**Figure 2:**
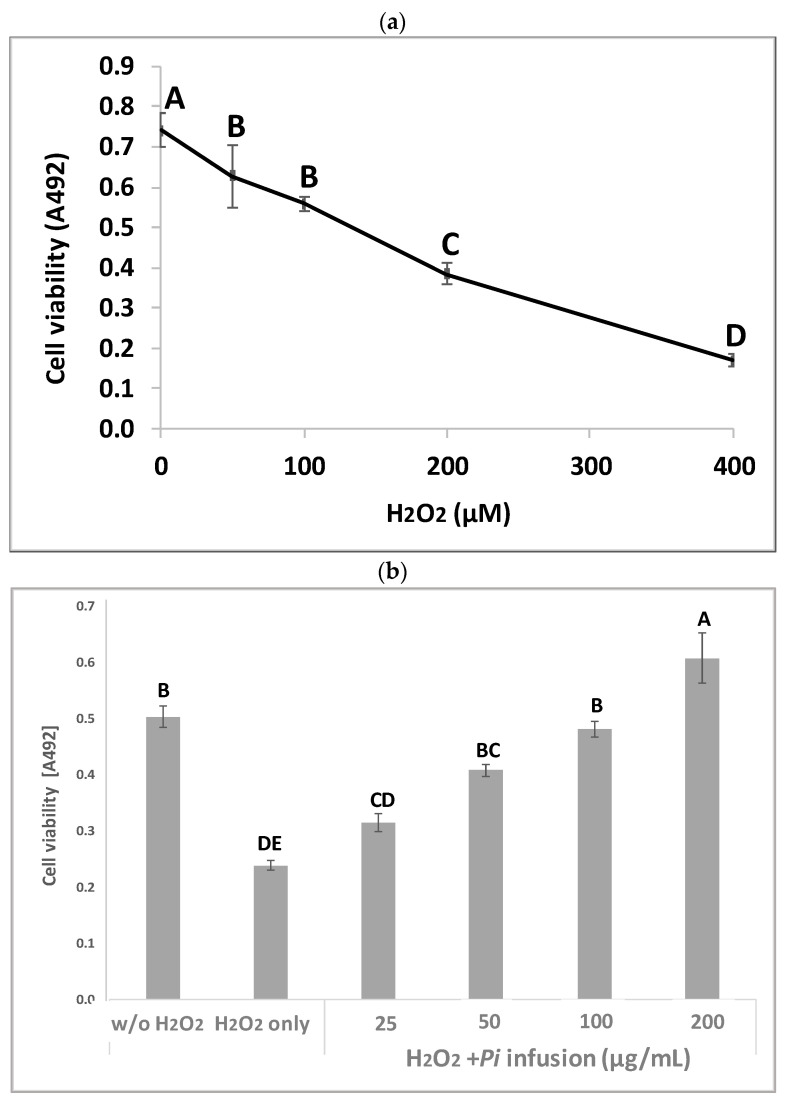

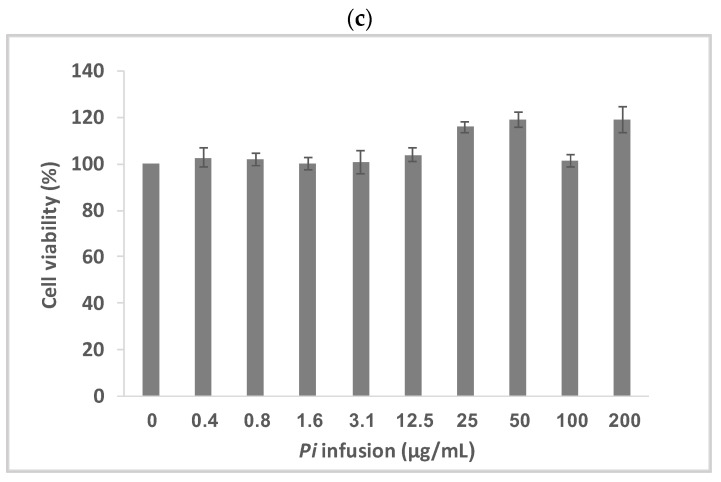
Neuroprotective effect of *Pulicaria incisa* (*Pi*) infusion against oxidative stress. (**a**) The cells were treated with increasing concentrations of hydrogen peroxide. Twenty hours later, cell viability was measured using the XTT assay. The results of untreated cells were defined as 100% viability. 0.745 OD = 100%. The results are presented as mean ± SEM and represent one experiment (out of two experiments) with 6 repetitions. The results were analyzed by one-way ANOVA followed by the Tukey–Kramer multiple comparisons test. Different letters (A, B, C, D) represent statistically significant differences (*p* < 0.001) (**b**) The cells were treated with increasing concentrations of *Pi* infusions for 2 h before exposure to 200 μM hydrogen peroxide. Twenty hours after the induction of the oxidative stress, cell viability was measured using the XTT assay. The results are presented as mean ± SEM and represent one experiment with 6 repetitions. The results were analyzed by one-way ANOVA followed by the Tukey–Kramer multiple comparisons test. Columns marked with different letters (A, B, C, D, E) represent statistically significant differences (*p* < 0.05). (**c**) Effect of *Pi* infusion on the viability of SH-SY5Y cells. Cells were treated with increasing concentrations of *Pi* infusions. Twenty-four hours later, cell viability was measured using the XTT assay. The results of untreated cells were defined as 100% viability. 0.364 OD = 100%. The data are expressed as mean ± SEM of two independent experiments (*n* = 12). The results were analyzed by one-way ANOVA. No significant differences were observed.

*Pi* infusion has antioxidant activity [8,9]. To determine the effects of *Pi* infusion on H_2_O_2_-induced neuronal cell death, the cytotoxicity of H_2_O_2_ in SH-SY5Y cells was first assessed using the XTT assay. Exposure of SH-SY5Y cells to H_2_O_2_ resulted in time- and concentration-dependent cell death (Figure 2a). The concentration of H_2_O_2_ selected for subsequent experiments (200 μM) resembled the concentration reported in rat striatum under ischemic conditions [27]. Pretreatment of the cells with *Pi* infusion before the induction of oxidative stress resulted in marked and statistically significant dose-dependent protection from cell death (Figure 2b). Of note, no *Pi* infusion cytotoxicity was recorded at any concentration tested (Figure 2c).

### 3.3. Pi Infusion Inhibits H_2_O_2_-Induced Caspase-3 Activity

**Figure 3:**
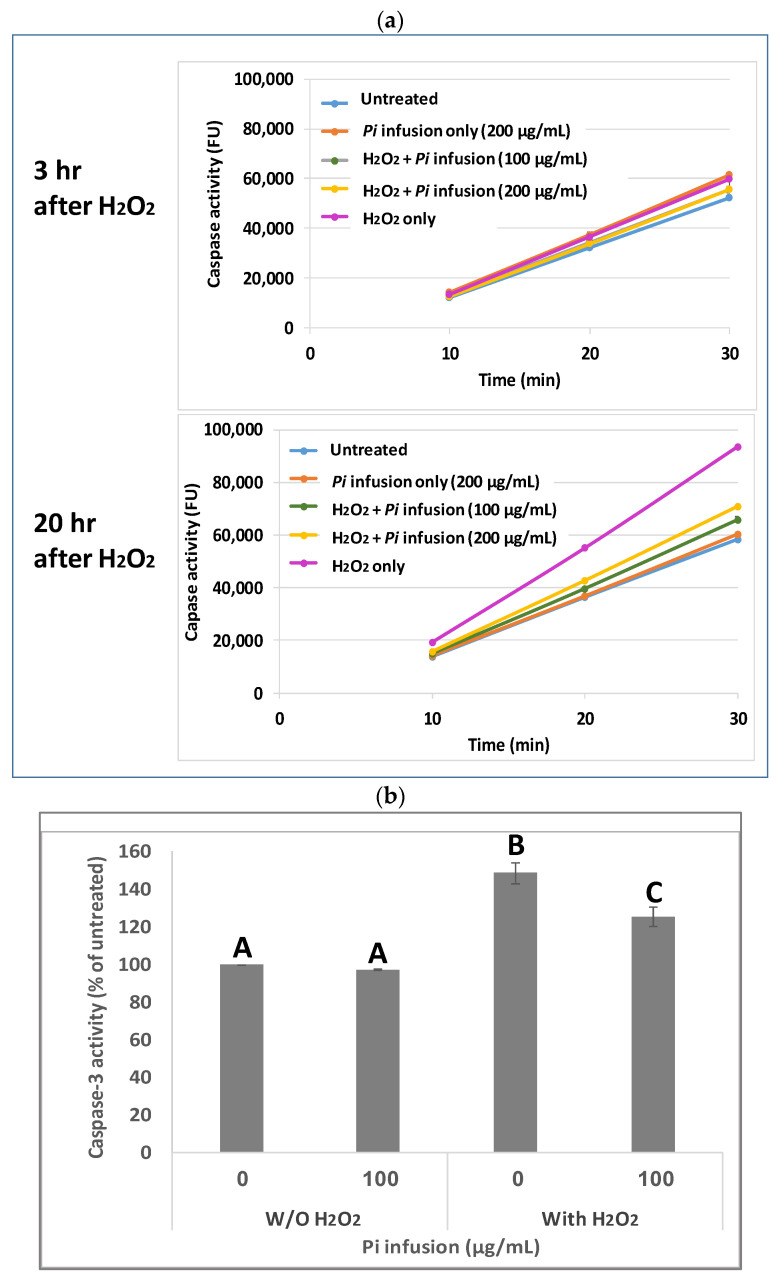
*Pi* infusion inhibits caspase 3 activity in neuronal cells exposed to hydrogen peroxide. Cells were pretreated with *Pi* infusion for 2 h before exposure to 200 µM H_2_O_2_. (**a**) Caspase 3 activity in cell lysates (20 µg) was measured (for 30 min) 3 h and 20 h after the induction of oxidative stress. The results are presented as mean ± SD of one experiment with two repetitions. (**b**) Caspase 3 activity in cell lysates (20 µg) was measured 20 h after the induction of oxidative stress for 30 min. The results are presented as mean ± SEM and represent three independent experiments (*n* = 6). The results were analyzed by one-way ANOVA followed by the Tukey–Kramer multiple comparisons test. Columns marked with different letters (A, B, C) represent statistically significant differences. (*p* < 0.05).

Caspases are cysteine proteases that are involved in cell death [28]. Since it has been previously shown that H_2_O_2_ activates caspase 3 during apoptosis [29,30], and since Pi infusion protects neuronal cells from oxidative-stress-induced cell death, we examined whether the neuroprotective mechanism of *Pi* infusion involves inhibition of caspase 3 activity. To this end, cells were pretreated with *Pi* infusion for 2 h before exposure to H_2_O_2_. The enzymatic activity of caspase 3 in cell lysates was then determined 3 h and 20 h after exposure to H_2_O_2_. Extended exposure to H_2_O_2_ led to a 1.6-fold increase in caspase-3 activity relative to untreated cells, while preincubation with 100 µg/mL Pi infusion before H_2_O_2_ exposure inhibited this activity by 79% (Figure 3a). After 3 h of exposure to H_2_O_2_, only a minor increase (1.1-fold) in caspase 3 activity relative to control untreated cells was recorded. At both time points, the levels of caspase 3 activity in cells treated with Pi infusion alone were very similar to that measured in untreated cells. Figure 3b presents the results of 20 h exposure to H_2_O_2_ which demonstrates that the activity of caspase 3 is significantly elevated by H_2_O_2_ (*p* < 0.001), and the induced activity is significantly (*p* < 0.01) inhibited (49% inhibition of the induced activity) by Pi infusion.

### 3.4. The Effect of Pi Infusion on H_2_O_2_-Induced ROS Levels in Neuronal Cells

**Figure 4:**
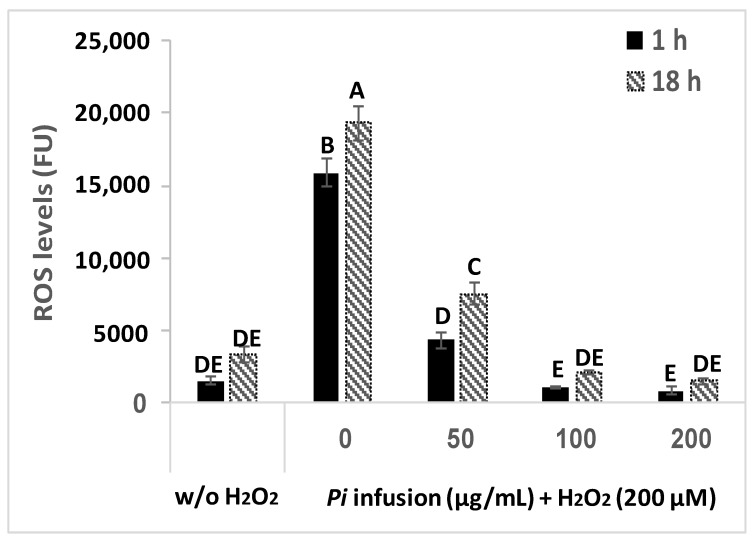
Pi infusion reduces H_2_O_2_-induced increases in intracellular reactive oxygen species (ROS) levels in neuronal cells. Cells were labeled with (2’7’-dichlorofluorescein diacetate) DCF-DA and washed, before being treated with increasing concentrations of Pi infusion for 2 h. Then, hydrogen peroxide (200 µM) was added to the medium and fluorescence levels were measured 1 h and 18 h thereafter. The results are presented as mean ± SEM and represent two independent experiments (*n* = 12). The results were analyzed by one-way ANOVA followed by the Tukey–Kramer multiple comparison test. Columns marked with different letters (A, B, C, etc.) represent statistically significant differences (*p* < 0.05).

H_2_O_2_ induces an elevation in intracellular ROS levels [9]. To assess the possibility that *Pi* infusion protects neuronal cells from H_2_O_2_-induced cell death by inhibiting the H_2_O_2_-induced elevation of ROS levels, changes in intracellular levels of ROS were measured with the ROS indicator DCF-DA. Treatment of cells with H_2_O_2_ (200 µM) resulted in a significant 10-fold and 6-fold elevation in intracellular ROS levels after 1 h and 18 h of treatment, respectively (Figure 4), suggesting that elevation in ROS levels is an early event that persists for at least 18 h. When pretreated with various concentrations of *Pi* infusion 2 h before H_2_O_2_ application, the increase in H_2_O_2_-induced ROS levels was entirely abrogated (at 100 and 200 μg/mL), both after 1 h and 18 h of treatment with H_2_O_2_.

### 3.5. Pi Infusion Upregulates Cellular Levels of Nrf2 and Induces Its Translocation to the Nucleus

**Figure 5:**
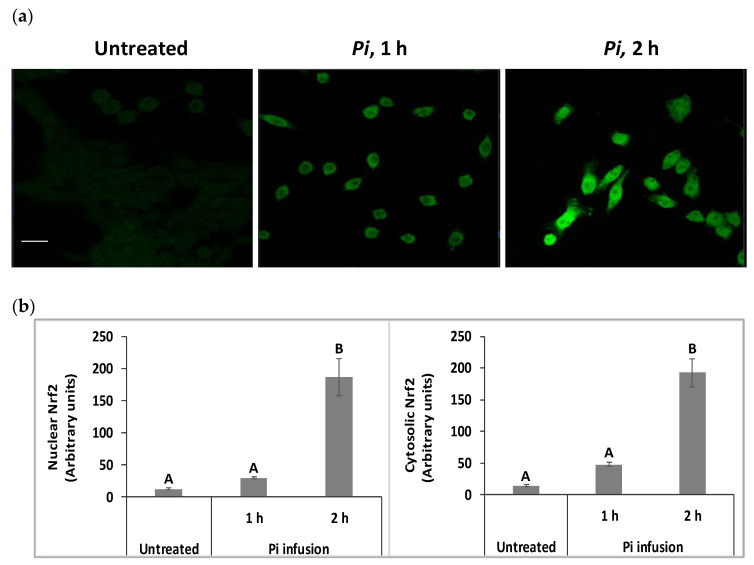
Pi infusion enhances nuclear factor erythroid 2–related factor 2 (Nrf2) expression levels and promotes Nrf2 translocation to the nucleus of neuronal cells. SH-SY5Y cells were treated by Pi infusion (200 µg/mL) for 1 h or 2 h and then immunostained for Nrf2 and nuclei were stained with DAPI. (**a**) Representative images of each treatment from two independent experiments. Each experiment was conducted in duplicates. Bar: 20 μm. (**b**) The Image J-measured fluorescence intensities of DAPI were used to normalize nuclear and cytoplasmic Nrf2 fluorescence intensity in 11 different photographed fields. Nrf2 intensity was normalized to DAPI intensity. The results are presented as mean ± SEM (*n* = 11) and were analyzed by one-way ANOVA followed by the Tukey–Kramer multiple comparison test. Columns marked with different letters (A, B) represent statistically significant differences (*p* < 0.05).

We have raised the possibility that Pi infusion activates an intracellular signal pathway in addition to its chemical antioxidant activity. The Nrf2 transcription factor is a key regulator of antioxidant responses and is known to be responsible for increasing the expression of antioxidant enzymes in the cell [31]. Under physiological conditions, most of the Nrf2 protein is located in the cytoplasm. However, following appropriate signals, including phytochemicals, Nrf2 translocates to the nucleus, where it activates the Nrf2-ARE pathway [32]. Both immunostaining and biochemical assays were used to test whether Pi infusion activates the Nrf2 pathway. Immunostaining with anti-Nrf2 and DAPI demonstrated that in control untreated cells, Nrf2 levels were relatively very low (Figure 5a). Following treatment with Pi infusion, cellular levels of Nrf2 were upregulated over time and underwent translocation from the cytoplasm to the nucleus. To quantify the effect of Pi infusion, the fluorescence intensities of Nrf2 in the nucleus and cytoplasm, were normalized relative to the DAPI intensity. Normalized Nrf2 fluorescence of Pi-treated (2 h of treatment) as compared to untreated cells were 14-, and 15-fold higher in the cytoplasm, and nucleus, respectively (Figure 5b).

**Figure 6:**
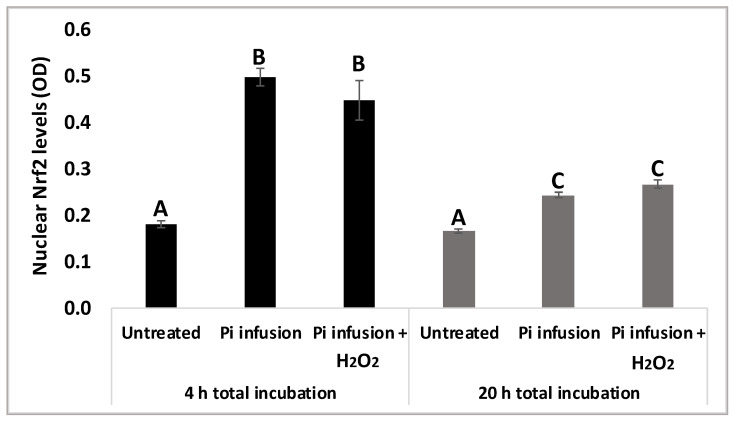
Pi infusion enhances Nrf2 expression levels in the nucleus of neuronal cells. SH-SY5Y cells were pretreated with Pi infusion (200 µg/mL) for 2 h or 18 h, and then incubated for an additional 2 h with H_2_O_2_ (200 µM). Nrf2 levels in the nucleus were then determined. The results are from a representative experiment out of two independent experiments, with each experiment conducted in duplicates. The results are presented as mean ± SEM (*n* = 4) and were analyzed by one-way ANOVA followed by the Tukey–Kramer multiple comparison test. Columns marked with different letters (A, B, C) represent statistically significant differences (*p* < 0.05).

To validate our results, protein levels of nuclear Nrf2 were assessed using a biochemical kit 4 h and 20 h following exposure to *Pi* infusion. Figure 6 shows that 4 h of treatment with *Pi* infusion increased nuclear Nrf2 levels 2.5-fold, relative to those of the control untreated cells. As expected, nuclear Nrf2 levels decreased with time, with Nrf2 levels after 4 h of *Pi* infusion treatment significantly higher than Nrf2 levels after 20 h of *Pi* infusion treatment. We further examined whether this effect was maintained in the presence of H_2_O_2_ by preincubating cells with *Pi* infusion for 2 h and then treating them with H_2_O_2_ for 2 h or 18 h. H_2_O_2_ had no significant effect on the Nrf2 levels that were induced by *Pi* infusion (Figure 6).

### 3.6. Pi Infusion Upregulates the Phosphorylation of the Transcription Factor Cyclic AMP Response Element-Binding Protein (CREB)

**Figure 7:**
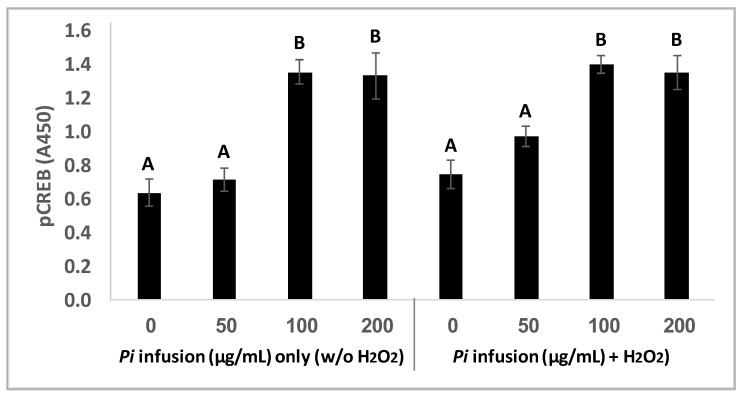
*Pi* infusion upregulates cyclic AMP response element-binding protein (CREB) phosphorylation. Cells were pretreated for 2 h with increasing concentrations of *Pi* infusion. H_2_O_2_ (200 µM) was then added, and pCREB levels in cell lysates (25 µg) were determined 30 min later. The results are presented as mean ± SEM of three experiments performed in duplicates (*n* = 6) and were analyzed by one-way ANOVA followed by the Tukey–Kramer multiple comparison test. Columns marked with different letters (A, B) represent statistically significant differences *p* < 0.001.

CREB is localized in the nucleus and acts as a transcription factor, which in its phosphorylated state mediates cellular response to a variety of signals [33]. CREB activity in neurons has been correlated with intracellular processes including survival, neurogenesis, and neuronal plasticity [33,34,35]. To gain more insight into the involvement of CREB signaling in the neuroprotective effect of Pi infusion, CREB phosphorylation was measured in neuronal cells treated with Pi infusion in the presence and absence of H_2_O_2_. Treatment of cells with Pi infusion for 2 h elicited a significant (*p* = 0.001) increase in CREB phosphorylation, with a maximal 2-fold elevation at 100 μg/mL and 200 μg/mL infusion (Figure 7) and did not change on the addition of H_2_O_2._ The optimal concentration for the induction of CREB phosphorylation was 100 μg/mL, which corresponded with the optimal concentration for cell protection from H_2_O_2_-induced cell death. It should be noted that treatment with Pi infusion and/or hydrogen peroxide, did not induce an increase in the levels of total CREB (data not shown).

### 3.7. Consumption of Pi Infusion Prevents Neuronal Cell Death in the Hippocampus of Aging Mice

**Figure 8:**
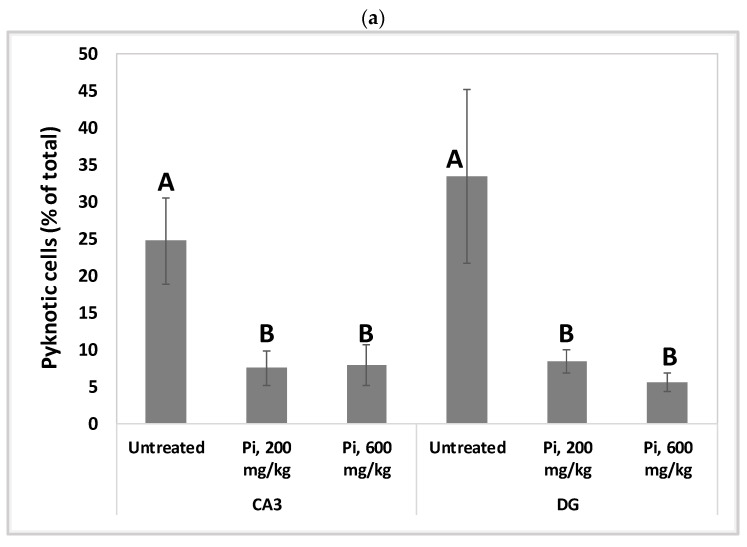

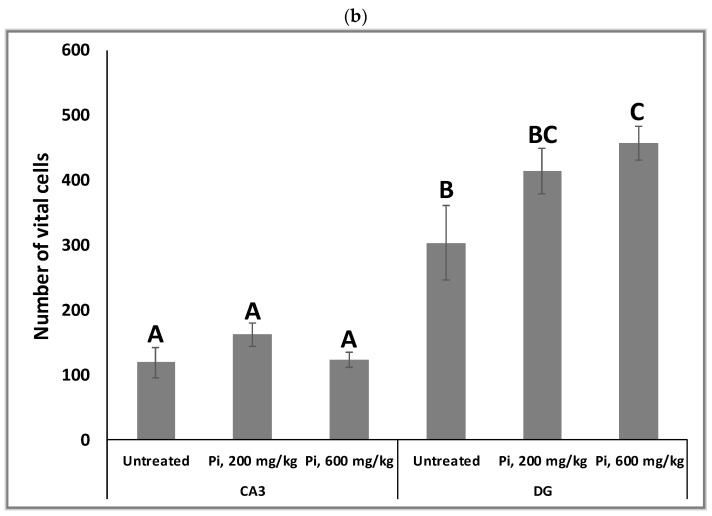
Consumption of *Pi* infusion enhances the viability of hippocampal neurons in aged mice. Brain sections of 19 months old mice treated daily for 18 months with *Pi* infusion, were stained with hematoxylin and eosin for morphometric analysis of the number of hippocampal neurons in the cornu Ammonis 3 (CA3) and dentate gyrus (DG) regions, as viewed through an objective magnification of X20. The percentage of pyknotic neurons was calculated as the number of pyknotic neurons out of the total number of neuronal cells (vital + pyknotic). The results are presented as mean ± SEM (*n* = 9). Columns marked with different letters (A, B, C) represent statistically significant differences *p* < 0.05.

Alterations in the intracellular redox ratio during aging appear to be highly localized to the brain hippocampus [36,37]. We, therefore, examined whether *Pi* infusion has a neuroprotective effect in vivo by preventing neuronal mortality in the hippocampus of aging mice. Figure 8a demonstrates that after 18 months of daily treatment with *Pi* infusion-supplemented drinking water, mouse brains contained a significantly reduced percentage of pycnotic cells in the CA3 and DG (70% and 83% reduction, respectively). In the DG area, 600 mg/kg *Pi* infusion also significantly enhanced the number of vital neuronal cells by more than 50% (Figure 8b).

## 4. Discussion

The increasing life span and number of patients with neurodegenerative diseases have brought to a growing interest in the development of nutritional supplements and functional foods and beverages that can support neuronal health. This study examined the effects of an infusion prepared from the *Pulicaria incisa* plant on cultured neuronal cells under oxidative stress and in vivo in aging mice. *Pi* infusions successfully protected neurons against oxidative stress, reduced intracellular ROS levels to the levels observed in control samples, increases Nrf2 expression, and induced its translocation from the cytoplasm to the cell nucleus.

Long-term oxidative stress, ROS accumulation, and oxidative damage to macromolecules in the brain are strongly associated with neurodegenerative diseases and are critical contributors to aging and age-related pathologies through organelle dysfunction (e.g., mitochondrial dysfunction), DNA damage, and protein aggregation. Nrf-2 is a redox-sensitive transcription factor that activates the antioxidant system in the cell [38] and upregulates genes that combat these adverse effects and mitigates age-related pathologies [1,39,40]. Nrf2 target genes protect against the development of age-related pathologies, both by neutralizing free radicals and by preventing the damage caused by ROS. Under basal conditions, Nrf2 is constantly degraded in the cytoplasm, but under oxidative stress, degradation is halted, leaving it stabilized and free to translocate to the nucleus, and subsequently activate the transcription of antioxidant genes whose promoters contain the antioxidant response element (ARE) [41]. Nrf2 induction prevents senescence and promotes proteasome formation and cell survival by preventing an increase in ROS, controlling the expression of anti-apoptotic mitochondrial proteins, such as BCL-2 and BCL-xL, and by modulating apoptosis and autophagy-related signaling [42,43].

The gradual reduction in Nrf2 expression with age resulting in increased oxidative stress appears to be a major risk factor for heightened susceptibility to neurodegenerative disorders and contributes to each of the hallmarks of aging, i.e., inhibited DNA repair, an overall increase in mutations and genomic instability, loss of proteostasis, telomere attrition, epigenetic alterations, mitochondrial dysfunction, deregulated nutrient metabolism and sensing, cellular senescence, stem cell exhaustion and altered intercellular communications [39,44,45,46,47].

The hippocampus has been reported to be among the three most susceptible areas to oxidative stress and consequently is the first to undergo a functional decline with age [36,37,48]. Several studies have suggested that pyramidal cells of CA3 and granule cells of the DG are oxidative stress-prone areas, whereas others have suggested that pyramidal cells of CA1 are more susceptible to oxidative damage [2]. The present study demonstrated that long-term consumption of *Pi* infusion can prevent neuronal cell death in the hippocampus during brain aging in mice.

Our observation that *Pi* infusion upregulates the levels of pCREB in neuronal cells can partially explain the neuroprotective effects of *Pi* infusion on neuronal cells under oxidative stress and in hippocampal neurons of aging mice. pCREB activates many genes, including genes that control brain development, function, and plasticity [33]. CREB is expressed in different regions of the brain, including the hippocampus [49]. Its levels decrease in aged rodent brains and dementia patients, with alterations and dysfunction in CREB signaling associated with cognitive deficits, as observed in normal aging and neurodegenerative diseases [50]. Therefore, CREB was suggested to be a therapeutic target for the treatment of age-related cognitive deficits [51].

Our in vivo results support the neuroprotective activities of the bioactive phytochemicals present in *Pi* infusion. These effects can be attributed to the presence of the central low-molecular-weight constituents of *Pi* infusion, i.e., aucubin (MW 346.33 g/mol), chlorogenic acid (354.31 g/mol) and quercetin (302.236 g/mol). Chlorogenic acid (~17.8% of the total constituents of *Pi* infusion) is a phenolic acid, which is an ester of caffeic acid and quinic acid [24]. It has been ascribed neuroprotective, anti-inflammatory, and antioxidant effects [24,52,53] and reported to prevent cognitive decline and neuronal damage in the hippocampus, and to protect against cognitive impairment in aged mice [54,55]. Chlorogenic acid was shown to regulate the oxidative stress-related Nrf2 pathway [56] and to stimulate the CREB pathway [53]. Quercetin is a flavonoid comprising ~8% of the total constituents of *Pi* infusion. Flavonoids have been implicated in neuronal proliferation and survival, by reducing oxidative stress and modulating various cellular signaling cascades, including CREB [57]. In vivo administration of purified flavonoids led to the phosphorylation of CREB and long-term potentiation in the hippocampus and had neuroprotective effects [57,58,59]. Aucubin is an iridoid glycoside (a class of monoterpenes) comprising ~2.8% of the *Pi* infusion. It has a wide range of pharmacological properties, including neuroprotective and anti-aging effects [60]. Aucubin provides a neuroprotective effect in primary cortical neurons and the hippocampus of experimental traumatic brain injury by inhibiting ROS generation, oxidative stress, and inflammatory responses [61]. The mechanism involves the activation of the Nrf2-induced antioxidant system [61,62,63]. It is still to be determined whether the effects of *Pi* infusion are elicited by a single constituent or if they are the output of the synergistic activities of several components.

## 5. Conclusions

The present study demonstrated that phytochemicals present in *Pi* infusion protect neurons from oxidative-stress-induced cell death in vitro and prevent neuronal cell death in the hippocampus of aging mice in vivo. We suggest that the protective effect of *Pi* infusion is mediated by the transcription factors Nrf2 and CREB. The presented results, along with previous evidence obtained in our laboratory [8,9], demonstrate the protective, anti-inflammatory, and antioxidant activity of *Pi* infusion on astrocytes and microglial cells. These position *Pi* infusion as a potential candidate for the regulation of oxidative stress and neuronal death in the brain during aging and neurodegenerative disease conditions.

## Figures and Tables

**Table 1:** Main compounds identified in the methanolic and aqueous phases of *Pulicaria incisa* infusion.

Compound	Rt	Total Peak Area (%) in the Methanolic Phase	Total Peak Area (%) in the Aqueous Phase
Malic acid	21.884		1.925
3-Aminophenol	25.702	1.414	
Citric acid	27.333	1.034	3.806
Quininic acid	28.051		1.754
Fructose	28.259	11.647	25.913
Talose	28.634	4.517	10.309
Glucose	28.646		9.038
Myo-Inositol	29.433	6.418	25.897
Quinoline, 5-chloro-8-ethoxy-7-iodo-	29.442		1.825
Galactaric acid	30.296		3.575
Caffeic acid	31.520	1.327	
Sucrose	37.814	15.348	8.261
Turanose	38.323		1.240
Aucubin	39.131	2.792	
Catechine (2*R*-*E*)	40.487	2.115	
Chlorogenic acid 1	41.573	16.192	
Quercetin	41.974	8.082	
Chlorogenic acid 2	42.115	1.424	

## Data Availability

The data presented in this study are available in the article.
